# Differential expression of DKK-1 binding receptors on stromal cells and myeloma cells results in their distinct response to secreted DKK-1 in myeloma

**DOI:** 10.1186/1476-4598-9-247

**Published:** 2010-09-16

**Authors:** Xiaoyi Dun, Hua Jiang, Jianfeng Zou, Jun Shi, Lili Zhou, Rong Zhu, Jian Hou

**Affiliations:** 1 Department of Hematology, Changzheng Hospital, the Second Military Medical University, Shanghai, China

## Abstract

**Background:**

The canonical Wnt signaling is concurrently important for osteoblast differentiation and myeloma cell proliferation. Its activation in myeloma cells and its inhibition in osteoblasts and their progenitors have been identified in the previous studies. Osteoblast progenitors and myeloma cells from a myeloma patient share the same bone marrow (BM) microenvironment, but respond differently to DKK-1 secreted by myeloma cells. The mechanisms remain unclear.

**Methods:**

Primary multiple myeloma (MM) cells were isolated from BM mononuclear cells of 12 MM patients. Human bone marrow stromal cells (SCs) were obtained from BM adherent cells of these MM patients and 10 healthy donors. The mRNA expression levels of DKK-1 binding receptor LRP5/6 and Kremen1/2 (Krm1/2) were analyzed by Real-time PCR in human myeloma cell line (HMCL) RPMI-8226, NCI-H929, U266, LP-1, CZ-1, KM-3, Sko-007, primary myeloma cells and SCs from 12 MM patients and SCs from 10 healthy donors. The binding capability of DKK-1 binding receptors to DKK-1 on primary myeloma cells and SCs was detected by flow cytometry assay.

**Results:**

The mRNA expression levels of DKK-1 binding receptor LRP5/6 and Krm1/2 in SCs from patients with MM were significantly higher than those in myeloma cells and in SCs from healthy donors. The binding capability to DKK-1of DKK-1 binding receptors on SCs from MM patients was obviously higher than those on myeloma cells and SCs from healthy donors by flow cytometry assay. Similar to the effects of coculture with rhDKK1, coculture of SCs from healthy donors with myeloma cells in the presence or absence of a Transwell insert did up-regulate SCs' mRNA levels of LRP5/6 and Krm1/2, and down-regulate their mRNA levels of β-catenin.

**Conclusion:**

Compared with myeloma cells, the SCs from MM patients overexpress DKK-1 binding receptors LRP5/6 and Krm1/2 in response to DKK-1 secreted by myeloma cells, which results in intracellular Wnt signaling inhibition. Our study provides a novel insight into mechanisms of myeloma associated osteolytic lesions.

## Background

Multiple myeloma (MM) is one of hematological malignancies characterized by bone marrow (BM) infiltration of monoclonal plasma cells and progression of osteolytic bone lesions [1]. It has been clearly identified that myeloma cells and osteolytic bone lesions are interdependent in that myeloma cells promote the development of the associated bone disease, and the microenvironment created by the resorbing bone in turn supports the growth and survival of myeloma cells, thus resulting in a vicious cycle of increased tumor burden and aggravating osteolytic bone disease [2,3]. MM associated bone loss is thought to be mediated by an uncoupled or imbalanced bone remodelling process with increased osteoclastic bone resorption and decreased osteoblastic bone formation [4]. It is only in the past few years that the suppression of osteoblastogenesis was found to contribute to the systemic bone loss and osteolytic bone lesions in myeloma associated bone disease besides the osteoclast activation [5]. The deficit of bone formation is attributable to osteoblastic dysfunction resulting from the proliferation of myeloma cells in the BM. However, the mechanisms by which myeloma cells affect the formation and function of osteoblasts are still under investigation.

Increasing evidences suggest that Wnt/β-catenin signaling activation has been linked to many human malignancies, including hematologic malignancies. It has been reported that MM cells have hallmarks of activated Wnt signaling [6]. In comparison with normal B-cell populations, MM cells express high levels of β-catenin, including the stabilized unphosphorylated form which functions in activating T-cell factor/lymphoid enhancer factor (TCF/LEF)-mediated transcription of target genes, thus stimulating the proliferation of MM cells [7].

Interestingly, the canonical Wnt pathway is essential to osteoblast differentiation and bone formation in addition to its role in tumor growth. Canonical Wnt signaling encourages osteoblast differentiation, proliferation and mineralization, while blocks apoptosis of osteoblasts [8]. The Wnt signaling antagonist dickkopf 1(DKK-1) has been shown to prevent Wnt-mediated terminal differentiation of mesenchymal stem cells (MSC) and osteoblast progenitors into osteoblasts. Furthermore, it has been reported that MM cells produce DKK-1, and a correlation between its expression by microarray analysis and the presence of MM associated bone lesions has been identified [9,10].

The role of Wnt signaling in MM seems to be a complexity allowing for its activation in myeloma cells and its inhibition in osteoblast progenitors. It is now well recognized that the interaction between tumors and their associated stroma can dramatically influence not only the tumor but also the behavior of the stroma. In the same tumor-stroma milieu, DKK-1 produced by MM cells could inhibit the Wnt signaling in osteoblasts and their progenitors, but failed to block the Wnt signal transduction in MM cells. Since the myeloma cells and the stromal cells (SCs) share the same BM microenvironment containing the ligand DKK-1, the determinant contributing to the different Wnt signal transduction may lie in the DKK-1 binding receptor expression.

The secreted protein DKK-1 interacts with the single-pass transmembrane proteins Kremen1/2 (Krm1/2) and the human low-density lipoprotein receptor-related protein 5/6 (LRP5/6) to form a ternary complex and to promote endocytosis and removal of LRP5/6 from the cell surface membrane, thereby inhibiting Wnt signaling [11,12]. In the present study, we first confirmed the DKK-1 protein expression in both MM cell lines and primary myeloma cells, and subsequently identified much lower β-catenin mRNA expression in SCs from patients with MM compared with those in primary myeloma cells and normal SCs, indicating the inhibition of Wnt signaling in patients' SCs in contrast to its activation in myeloma cells. Then we demonstrated that the mRNA expression levels and the DKK-1 protein binding capability of DKK-1binding receptors LRP5/6 and Krm1/2 in SCs from patients with MM were significantly higher than those in SCs from healthy donors and those in MM cells, and also found coculture with rhDKK-1 or MM cells in the presence or absence of a Transwell insert could up-regulate the mRNA expression of LRP5/6 and Krm1/2, and down-regulate β-catenin mRNA expression in normal human SCs. Thus, we concluded that, compared with myeloma cells in the same BM milieu, the SCs overexpressed DKK-1 binding receptor in response to DKK-1, which results in their intracellular Wnt signaling inhibition.

## Materials and methods

### Reagents and Cytokines

Recombinant human DKK-1(rhDKK-1) was purchased from R&D Systems (Minneapolis, MN, US). pcDNA3.1(-)-DKK-1 vector was a generous gift from Dr Junzhou Niu, the fourth Military Medical University(Xian, China). Unless stated otherwise, all other chemical and tissue culture reagents were purchased from Sigma Aldrich Chemical Company (St. Louis, MO, US).

### Patients

We studied 12 hospitalized patients with newly diagnosed MM [13] between October 2007 and January 2009 in our department. The clinical features are shown in Table 1. In addition, 10 healthy donors (6 males and 4 females) with the median age of 60 years, ranging from 40 to 72, were included in the study. Each healthy donor was examined to ensure that there was no evidence of bone disease (osteoporosis or osteoarthritis). Bone marrow aspirates were obtained from the iliac crest of all patients with MM and healthy donors at diagnosis after obtaining informed consent according to the Declaration of Helsinki and our local ethics committee. The approval of the study was obtained from the Second Military Medical University for Medical Science Institutional Review Board.

**Table 1:** Clinical features of MM patients

No. patients	12
Gender	7M-5F
Age median (Range)	61(35-76)
Type of M-protein	IgG(5), IgA(3), IgD(1).
	Light chain(2), non-secretory(1)
Stage at diagnosis (Durie-Salmon)	Stage IIA: 3(25%)
	Stage IIIA:7 (58.3%)IIIB: 2(16.7%)
Bone disease status at diagnosis	no osteolytic lesions:2(16.7%)
	one to three lytic bone lesions:4(33.3%)
	more than three lytic lesions:6(50%)

### Cells and Cell Culture Conditions

#### Cell lines

Human myeloma cell lines (HMCLs) RPMI-8226, NCI-H929, U266, LP-1, CZ-1, KM-3, Sko-007 were used in this study. NCI-H929 was introduced from Dr. Margaret H.L. Ng (Prince of Wales Hospital, Chinese University of Hong Kong). RPMI-8226 (CCL-155™), Sko-007(CRL-8033-1™) and U266 (TIB-196™) were obtained from American Type Culture Collection (ATCC). LP-1 secreting IgG λ light chain was kindly provided by Dr. Hallek (Laboratorium für Molekulare Biologie, Genzentrum, Ludwig-Maximilians-Universität München, Germany). CZ-1 secreting λ light chain was established from the bone marrow of an advanced stage patient with MM classified as λ light chain type in our laboratory [14]. KM-3, a non-secreting MM cell line, was conserved in our own laboratory. All the cell lines used in this study were stored in liquid nitrogen in our laboratory. Before experiments, cells were immediately cultured after thawing in RPMI 1640 medium (Sigma-Aldrich, St. Louis, MO, USA) containing 10% fetal bovine serum, 200 units/ml penicillin, 200 g/ml streptomycin, minimal essential vitamins, sodium pyruvate, and glutamine.

#### Primary human multiple myeloma cells and SCs

Primary multiple myeloma cells were isolated from BM mononuclear cells (BMMNC) of multiple myeloma patients at diagnosis by immunomagnetic beads using anti-CD138 monoclonal antibody (mAb)-coated microbeads (MACS, Miltenyi Biotec). Only samples with purity >90%, checked by flow cytometry, were used immediately after purification in the study.

Human BM SCs (HMSC) were obtained from primary BM adherent cells after an attachment period and cultured in Dulbecc's Modified Eagle Medium(DMEM), containing 15% heated-inactivated FBS, penicillin (100 U/ml), streptomycin (100 mg/ml), and 4 mM L-glutamine.

#### Cell cocultures

A series of coculture experiments were done with HMCL RPMI-8226 or NCI-H929 (1 × 10^6^cells) and confluent HMSC of healthy donors (5 × 10^5^cells). Myeloma cells were added directly to the cultures or placed in a Transwell insert (Corning Biotec) in RPMI 1640 with 10% FCS for 24 to 72 h. At the end of culture period, cocultures were depleted of myeloma cells using a negative immunoselection with anti-CD138 mAb (MACS, Miltenyi Biotec, Bergisch Gladbach, Germany).

In selected experiments, HMSC of healthy donors (5 × 10^5^cells) were incubated in the presence of rhDKK-1 (100-700 ng/mL) for 48 to 72 h.

#### Real-time PCR

Total RNA was isolated using TRIzol reagent (Invitrogen, Calsbad, CA). Quantitative PCR (qPCR) was performed using an ABI Prism 7500 sequence detection system (Applied Biosystems, Foster City, CA). Primers, including LRP5/6, Krm1/2 and β-catenin were designed using the ABI Primer Express 2 software. Primer sequences are: LRP5, 5- GGGCCACTCTGGCTTCTCT -3(forward) and 5- CATCACAGTTCACATTTCTCATGTTT -3(reverse); LRP6, 5-GTGAGAGAAGAGAACGCGAGAAG-3(forward) and 5- GTCCCGTCTGTTTGCATAAAGC -3(reverse); Krm1, 5- CAGCCCCGATGCATCCT -3(forward) and 5- TCCGCGTATAAGTGCTTGTGA-3(reverse); Krm2, 5- TGGCGCTACTGCGACATC -3(forward) and 5-GTCCACAAAGCATCCCAGGTA -3(reverse); β-catenin, 5- TGCCATTCCACGACTAGTTCAG -3(forward) and 5- CGTACGGCGCTGGGTATC-3(reverse). Using Reaction Ready First-Strand cDNA Synthesis kit (Toyobo Biotec), 1 μg total RNA was reverse transcribed and then real-time PCR was done by adding cDNA directly to PCR Master Mix containing SYBR Green and References Dyes (Toyobo Biotec). The mixtures were then aliquoted into 96-well PCR array plates, which profile the expression of LRP5/6, Krm1/2 and β-catenin plus controls. Each cDNA sample was analyzed in triplicate in parallel with GAPDH as a control. Thermal cycling was done according to the manufacturer's protocol (Ta, 60°C; total cycles, 40-45). Finally, we determined the fold changes in expression byΔΔCt as described previously [15].

### Western Blot Analysis

Total cell lysates for Western blots were obtained using a commercial kit (Sigma Chemical). Lysates were separated by sodium dodecyl sulfate-polyacrylamide gel electrophoresis (SDS-PAGE), transferred to polyvinylidene difluoride membranes, and then blocked in TBS plus 5% BSA and 0.1% TWEEN for 1 hour, before incubating with anti-DKK-1 (Milipore, Bedford, MA). Anti-β-actin (Sigma-Aldrich, St.Louis, MO, USA) was used as control.

### Transfectants, expressions and purification

To generate a DKK-1 expressing cell line, 293T cells were transiently transfected, using LipofectAMINE™2000 reagent (Invitrogen, Carlsbad, CA), with a pcDNA3.1(-)-DKK-1 vector carrying a DKK-1 cDNA, according to manufacturer's instructions. In the supernatant, His-DKK-1 fusion protein was collected. With the Ni-Agarose His protein purification kit, DKK-1 fusion protein was purified. Using anti-DKK-1 antibody (Milipore, Bedford, MA) and anti-His antibody (Sigma Aldrich, St. Louis, US), His-DKK-1 fusion protein in the supernatant was identified by Western blotting analysis.

### Flow cytometry

Flow cytometry (FCM) was adopted to determine the expression level of DKK-1-binding receptors on the myeloma cells and SCs. Primary myeloma cells and SCs from MM patients and healthy donors (2 × 10^5^cells) were treated with different concentrations of recombinant His-DKK-1 fusion protein for 30 minutes on ice, washed 2 times and resuspended in phosphate-buffered saline (PBS). Then, fluorescein isothiocyanate (FITC)-labeled anti-6×His polyclonal antibodies (Abcam) were added to cell suspensions, incubated for 30 minutes on ice, and washed 3 times before analysis. Samples were analyzed using a flow cytometer (Beckman Coulter, Fullerton, CA). Cell suspensions without FITC-labeled anti-6×His polyclonal antibodies were used as negative controls. Mean fluorescence intensity (MFI) is used as index of detection.

### Statistical analysis

Statistical significance was analyzed by one-way ANOVA using SPSS version 13.0 software and considered significant given *P *less than or equal to 0.05. Data were presented as means ± SEM unless otherwise stated.

## Results

### Expression of DKK-1 protein by human MM cells

**Figure 1:**
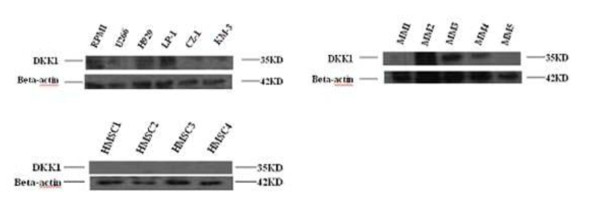
**Expression of DKK1 protein by human multiple myeloma cells**. DKK1 protein expression was evaluated by Western blot analysis in cell lysates from HMCLs, purified CD138+ multiple myeloma cells and SCs of multiple myeloma patients. DKK-1 protein expression was obviously expressed in RPMI-8226, NCI-H929 and LP-1 and weakly expressed in U266, CZ-1 and KM-3. DKK-1 protein expression was positive in 3 of the 5 primary CD138+ myeloma cells and negative in all the SCs from 4 patients with MM. (HMCLs: RPMI-8226, U266, NCI-H929, LP-1, CZ-1, KM-3. MM1-5: freshly purified CD138+ myeloma cells of 5 MM patients. HMSC1-4: Human BM SCs from 4 MM patients)

We examined the expression of DKK-1 protein in 6 HMCLs, and 5 freshly purified primary CD138+ myeloma cells and 4 SCs from patients with MM by western blotting. It showed that DKK-1 protein was obviously expressed in RPMI-8226, NCI-H929 and LP-1, and weakly expressed in U266, CZ-1 and KM-3. DKK-1 protein expression was positive in 3 of the 5 primary CD138+ myeloma cells and negative in all the SCs from 4 patients with MM (Fig. 1).

### mRNA expression of LRP5/6, Krm1/2 and β-catenin in HMCLs, CD138+ primary myeloma cells and SCs from patients with MM and healthy donors

**Figure 2:**
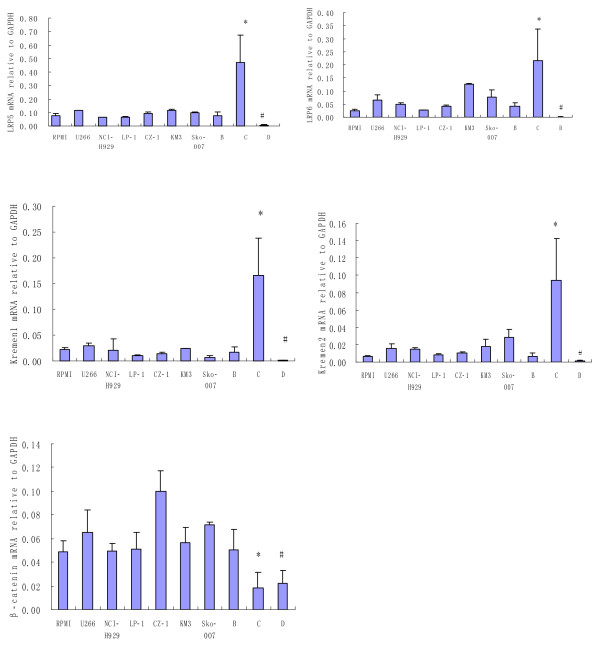
**LRP5/6, Krm1/2 and β-catenin gene expression of HMCLs, CD138+ primary myeloma cells and SCs from patients with MM and healthy donors**. HMCLs include RPMI-8226, NCI-H929, LP-1, U266, CZ-1, KM-3 and Sko-007. B: CD138+ primary myeloma cells. C: SCs from patients with MM. D: SCs from healthy donors. LRP5/6 and Krm1/2 mRNA expression in SCs from patients with MM (C) were significantly higher than those in SCs from healthy donors (D) (p < 0.001) and those in CD138+ primary myeloma cells (B) (p < 0.01). β-catenin mRNA expression in HMCLs and CD138+ primary myeloma cells was obviously higher than those in SCs from patients with MM and healthy donors (p < 0.05). There was no significant differences in the β-catenin mRNA expression levels between SCs from healthy donors and those from patients with MM (p > 0.05).

LRP5/6 and Krm1/2 mRNA expression levels in SCs from patients with MM (C) were significantly higher than those in SCs from healthy donors (D) (p < 0.001) and those in CD138+ primary myeloma cells (B) (p < 0.01). β-catenin mRNA expression levels in HMCLs and CD138+ primary myeloma cells were obviously higher than those in SCs from patients with MM and healthy donors (p < 0.05). There were no significant differences in the β-catenin mRNA expression levels between the SCs from healthy donors and those from patients with MM (p > 0.05) (Fig. 2).

### Influences of MM cells on the mRNA expression of LRP5/6, Krm1/2 and β-catenin in SCs from healthy donors in coculture

**Figure 3:**
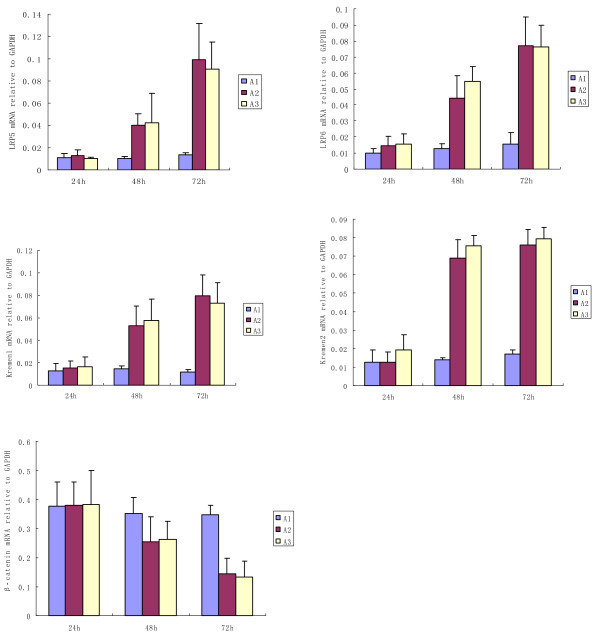
**Effects of MM cells on the mRNA expressions of LRP5/6, Krm1/2 and β-catenin in SCs from healthy donors in coculture with RPMI-8226**. A1: SCs from healthy donors. A2: SCs from healthy donors in coculture with RPMI-8226 in the absence of a Transwell insert. A3: SCs from healthy donors in coculture with RPMI-8226 in the presence of a Transwell insert. LRP5/6, Krm1/2 and β-catenin gene expressions were detected in SCs from healthy donors after 24h-72 h coculture with RPMI-8226 in the presence or absence of a Transwell insert. No significant effect on the gene expression was observed after 24 h coculture. After 48 h, a significant up-regulation of LRP5(fold change, 3.5), LRP6 (fold change, 4), Krm1(fold change, 4) and Krm2 (fold change,5) together with an down-regulation of β-catenin (fold change, **-**1.3) in SCs from healthy donors were seen whether in the presence or absence of a Transwell insert. After 72 h, mRNA expression of LRP5(fold change, 7), LRP6 (fold change, 5), Krm1(fold change, 7) and Krm2 (fold change, 4.5) were elevated, meanwhile mRNA expression of β-catenin (fold change, -2.7) was decreased.

The mRNA expressions of LRP5/6, Krm1/2 and β-catenin were detected in SCs from healthy donors after 24-72 h of coculture with RPMI-8226 or NCI-H929 in the presence or absence of a Transwell insert. We found a significant up-regulation of LRP5 (fold change, 3.5/7), LRP6 (fold change, 4/5), Krm1 (fold change, 4/7) and Krm2 (fold change, 5/4.5) together with the corresponding down-regulation of β-catenin (fold change, **-**1.3/-2.7) in SCs from healthy donors after 48h/72 h coculture with RPMI-8226 in the presence or absence of a Transwell insert. No significant influence on the gene expression was observed at 24 h after coculture (Fig. 3). Similar results were obtained with NCI-H929 in coculture (data not shown).

### Effects of rhDKK-1 on the mRNA expression of LRP5/6, Krm1/2 and β-catenin in SCs from healthy donors in coculture

**Figure 4:**
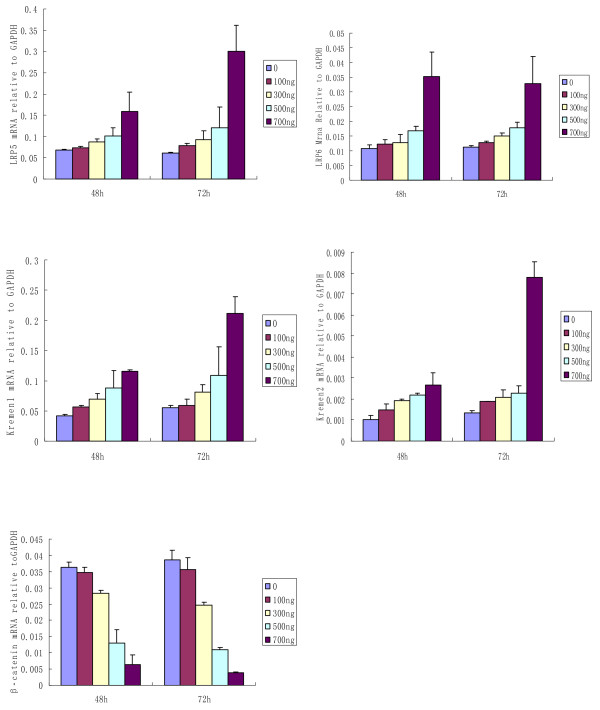
**Effects of rhDKK1 on the mRNA expression of LRP5/6, Kremen1/2 and β-catenin in SCs from healthy donors in coculture**. The mRNA expression of LRP5/6 and Krm1/2 were significantly elevated and the mRNA expression of β-catenin was decreased in SCs from healthy donors after 48h-72 h of coculture with rhDKK1 at concentration of 700 ng/ml.

The mRNA expressions of LRP5/6, Krm1/2 and β-catenin were examined in SCs from healthy donors after 48-72 h of coculture with rhDKK-1 at the concentrations ranging from 100 to 700 ng/ml. The mRNA expression levels of LRP5/6 and Krm1/2 in SCs from healthy donors were time-dependently and dose-dependently elevated after the coculture with rhDKK-1. Correspondingly, the mRNA expressions of β-catenin in SCs significantly decreased in a dose-dependent manner (Fig. 4).

### The quantitative analysis of DKK-1 binding receptors on the myeloma cells and SCs

**Figure 5:**
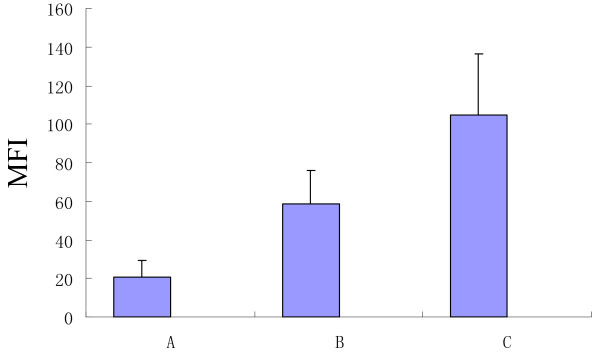
**The quantitative analysis of DKK1 binding receptors on the cell surface of myeloma cells and SCs**. The mean fluorescence intensity (MFI) of the DKK-1 binding receptors on myeloma cells (B), SCs from healthy donors (A) and patients with MM (C) were (58.6 ± 17.2), (20.8 ± 8.4), (104.5 ± 32.1), respectively. There were significantly difference among three groups(P < 0.05).

Flow cytometry results showed that recombinant His-DKK-1 fusion protein could specifically bind the DKK-1 binding receptors on the myeloma cells and SCs. The mean fluorescence intensity (MFI) of the DKK-1 related receptors on myeloma cells (B), SCs from healthy donors (A) and patients with MM (C) were (58.6 ± 17.2), (20.8 ± 8.4) and (104.5 ± 32.1), respectively (Fig. 5). There were significant differences among the three groups (P < 0.05).

## Discussion

MM is a debilitating and incurable malignancy of antibody-secreting plasma cells that predominantly accumulate in the BM. The tropism of myeloma cells for BM suggests that BM provides a unique microenvironment favoring the growth and survival of MM cells. The interaction between myeloma cells and osteoclasts that contributes to the tumor growth and the aggravation of osteolytic lesions has been well recognized. The recent discovery of the functional defect of osteoblasts in myeloma indicates the importance of osteoblasts in the lytic process [8].

Canonical Wnt signaling is extremely required in osteoblastogenesis and normal bone metabolism, and secretion of Wnt signaling antagonists by MM cells is thought to contribute to the bone-destructive process as well as disease progression by inhibiting the differentiation and maturation of osteoblasts. The positive correlation between the DKK-1 expression and the osteolytic lesions has been identified [16]. It has been shown that primary myeloma cells from osteolytic MM patients significantly overexpressed the Wnt inhibitor DKK-1 in comparison with the plasma cells from patients with monoclonal gammopathy of undetermined significance (MGUS) and normal plasma cells. In the present study, we demonstrated that most of the HMCLs and the primary myeloma cells produced DKK-1 protein, consistent with the previous reports [17].

It is definite that Wnt signaling is activated in myeloma cells, and the key signaling molecule β-catenin accumulates in the nuclei and activates the transcription of its target genes [18]. On the contrary, blockade of Wnt signaling in MSCs by DKK-1 has been shown to result in suppression of osteoblastic bone formation which contributes to the systemic bone loss and osteolytic bone lesions in myeloma [19]. Our results showed that β-catenin expression levels in myeloma cells were considerably higher than those in SCs, and that β-catenin expression levels in SCs from patients with MM were even a little lower than those from healthy donors, suggesting that Wnt signaling is highly inhibited in SCs from patients with MM. Given the fact of osteoclast activation and the consequent enhanced bone resorption in MM, the inhibition of Wnt signaling in SCs is considered to contribute to attenuated bone formation and aggravated imbalance of bone metabolism.

Though in the same tumor-stroma milieu, DKK-1 produced by MM cells could inhibit the Wnt signaling in osteoblast progenitors, but failed to block the Wnt signal transduction in MM cells. The discrepency in the condition of canonical Wnt signaling between SCs and myeloma cells in response to DKK-1 drove us to investigate the expression of DKK-1 binding receptor on the cell surface.

Krm was originally discovered as a novel transmembrane protein containing the kringle domain. Krm1/2 is high-affinity receptors for DKK. DKK-1 and the Wnt coreceptor, LRP5/6, interact with Krm1/2 to form a ternary complex and inhibit Wnt/beta-catenin signaling [20]. In the present study, we found the gene expression levels of LRP5/6 and Krm1/2 in SCs from patients with MM were much higher than those of primary myeloma cells, and those of normal SCs. The differential expression of DKK-1 binding receptors on the surface of SCs and myeloma cells probably lead to their diverse Wnt signal transduction. More importantly, the results are further confirmed by our subsequent study on DKK-1 protein binding capability of these DKK-1 binding receptors. We used recombinant His-DKK-1 fusion protein and FITC-labeled anti-His polyclonal antibodies to determine the expression and binding capacity of DKK-1 receptor protein on the surface of myeloma cells and SCs. It was demonstrated that the amount of DKK-1 binding receptor protein on the SCs from patients with MM were much higher than those on the primary myeloma cells and on the SCs from healthy donors. Mao et al. [11] found that DKK-1 binds readily to cells overexpressing LRP5/6, but not cells overexpressing Wnt or Frizzled. Compared with the myeloma cells in the same BM microenvironment, SCs from patients with MM overexpressed LRP5/6 and Krm1/2, and thus had higher affinity to DKK-1, contributing to the different response to DKK-1.

In our study, SCs from healthy donors were cocultured with HMCLs, RPMI-8226 and NCI-H929. We found significant upregulation of LRP5/6 and Krm1/2 mRNA expression and down-regulation of β-catenin mRNA expression in SCs in similar degrees in the presence or absence of a Transwell insert after 48-72 h coculture. Furthermore, similar results in the regulation of LRP5/6, Krm1/2 and β-catenin were also found in SCs from healthy donors after the treatment of rhDKK-1 at a high dose. It has also been identified in our study that both RPMI-8226 and NCI-H929 secrete DKK-1 protein. All of these results mightily suggest that myeloma cells could promote expressions of LRP5/6 and Krm1/2 on SCs in coculture via DKK-1 secretion. Likewise, the highly expressed DKK-1 binding receptors on the SCs from MM patients in our study probably result from the DKK-1 secretion by myeloma cells in the same BM milieu. Noteworthy, rhDKK-1 can induce the foresaid alterations of gene expressions only at a high concentration (700 ng/ml) in our study, in accordance with the earlier reports that DKK-1 inhibited the differentiation of SCs/osteoprogenitor cells to osteoblast only at high concentrations [21].

Taken together, our study elucidated the mechanism by which myeloma cells and SCs in the same BM microenvironment respond differently to DKK-1 produced by MM cells. In comparison with myeloma cells, SCs from MM patients overexpress DKK-1 binding receptors LRP5/6 and Krm1/2, which probably lead to the blockade of intracellular Wnt signaling in SCs. Our study provides a novel insight into mechanisms of myeloma associated osteolytic lesions.

## Competing interests

The authors declare that they have no competing interests.

## Authors' contributions

XD participated in the design of study, carried out the molecular studies, performed the statistical analysis and drafted the manuscript. HJ carried out the experiment of flow cytometry and revised the manuscript. JZ participated in the experiment of magnetic cell sorting. JS carried out the experiment of cell culture. LZ participated in the design of study. RZ performed the statistical analysis. JH conceived of the study and participated in its coordination.

All authors read and approved the final manuscript.
